# Environmental cues signal through circadian control of sarcomeric gene expression during cardiomyocyte hypertrophy

**DOI:** 10.1080/15592294.2026.2694794

**Published:** 2026-07-22

**Authors:** Adrian Arrieta, Alexa Jennings, Pratiti Dasgupta, Natalie D. Gehred, Tatiana Gromova, Erik Sandoval, Kunhua Song, Mansoureh Eghbali, Matthew A. Fischer, Thomas M. Vondriska

**Affiliations:** a Department of Anesthesiology & Perioperative Medicine, David Geffen School of Medicine at UCLA, Los Angeles, California, United States; b Department of Medicine, Division of Cardiology, David Geffen School of Medicine at UCLA, Los Angeles, California, United States; c Department of Physiology, David Geffen School of Medicine at UCLA, Los Angeles, California, United States; d Molecular Biology Institute David Geffen School of Medicine at UCLA, Los Angeles, California, United States; e Internal Medicine, Heart Institute, Center for Regenerative Medicine University of South Florida, Tampa, Florida, United States

**Keywords:** Circadian, chromatin, myocyte, sarcomere, hypoxia

## Abstract

Environmental disruption alters circadian clock gene expression, increasing the risk of adverse cardiac events and suggesting cardiomyocyte-specific circadian responses to external stimuli. Analyses of previously reported transcriptomic data revealed increased expression of Titin-cap (Tcap) in adult compared to embryonic myocytes and identified myosin light chain 2 (Myl2) as clock-controlled. Given cardiac sarcomeric roles of the encoded proteins, we hypothesized that extracellular cues driving postnatal cardiac maturation and hypertrophy influence time-of-day Tcap and Myl2 expression. Tcap induction was concomitant with neonatal myocyte binucleation, fetal gene suppression, and increased heart weight during the early phase of cardiac growth. Since norepinephrine stimulates β-adrenergic and α-adrenergic receptors, the latter driving clock-controlled transcriptional remodeling, phase‑response curves of the β‑adrenergic agonist isoproterenol (ISO) following α-adrenergic stimulation with phenylephrine (PE) were performed on neonatal rat ventricular myocytes (NRVM), revealing periodic myocyte hypertrophy and TCAP protein expression. PE entrenched ISO-mediated Tcap suppression, to which oscillatory Per2 and Myl2 transcription was impervious. Differential NRVM culture density revealed biomass-dependent changes in Per2 and Tcap transcription and hypertrophy timing. Hypoxia initiated myocyte atrophy and Bmal1-dependent Tcap transcription, reflected in decreased heart weight and increased Tcap expression in hypoxic neonatal rat hearts. Tcap depletion impaired Bmal1 and fetal hypertrophic gene expression, compromised hypoxia-mediated Myl2 transcriptional suppression, and aggravated hypoxia-induced atrophy. In summary, Tcap and Myl2 are circadian genes differentially influenced by environmental factors, including adrenergic stimulation, paracrine signaling, and O_2_ tension during postnatal cardiac maturation. These findings have implications for the distinct regulation of myocyte growth and maturation, by external cues during the postnatal period.

## Introduction

Circadian clocks function throughout the eukaryotic world to control rhythmic behaviors of multi-cellular organisms. The molecular clock operates at the cell and organ level, underpinned by a subcellular network of transcriptional and protein circuits that operate independently of, but also in response to, cues emanating from the extracellular environment [1]. With changes in these external cues, clock-controlled genes can display changes in the timing or amplitude of their expression and affect downstream biological outputs, including metabolism, proliferation, and cell–cell communication [2].

The circadian clock also transcriptionally controls tissue- and cell type-specific processes, and in the heart this is exemplified by clock-controlled, oscillatory expression of the gene encoding the sarcomeric protein Titin-cap (TCAP) [3,4], amongst other genes that contribute to muscle cell differentiation and development. TCAP (a.k.a. telethonin) is a z-disc protein that contributes to sarcomere integrity via tethering of sarcomeric proteins, including titin and α-actinin, to this region [5]. Mutations in TCAP can cause skeletal- or cardiomyopathy, potentially by altering such tethering [6,7]. Germline TCAP knockout mice are normal at baseline but show worsened ventricular dilation and impaired ejection fraction following pressure‑overload stress [5]. As an anchor protein, TCAP participates in the formation and maintenance of the tension and stretch sensory machinery at the sarcomere [8] and interacts with p53 to suppress its pro-apoptotic transcriptional activity [5]. This suggests that TCAP is not required for sarcomerogenesis, is required for sarcomere adaptability to conditions that result in mechanical strain and may mediate bidirectional nuclear-sarcomeric signaling. While the functional importance of TCAP and its control by the circadian clock have been reported, the precise coupling of environmental cues—which influence cardiac function at baseline and during disease—to circadian-controlled sarcomeric protein expression and function is unknown.

Circadian regulation of genes encoding sarcomeric proteins is of critical importance, given the impact of time-of-day on precipitating acute cardiovascular events and the endogenous fluctuations in cardiac hormones, such as catecholamines, which may contribute to this phenomenon [9]. We have previously demonstrated that depletion of the master circadian rhythm transcription factor, Bmal1, in cultured cardiac myocytes impairs histone turnover and transcription of cardiac-specific clock-controlled genes, including Tcap. Here, we investigate how expression of Tcap, an established striated‑muscle-specific clock-controlled gene, and ventricle-specific myosin light chain 2 (Myl2/Mlc2v), a newly identified clock-controlled gene, change during postnatal growth of the heart, and in response to external cues that influenced postnatal cardiac maturation: adrenergic signaling, paracrine signaling, and O_2_ tension. These findings reveal novel insights into clock control of sarcomeric gene expression in myocytes and have implications for the design and implementation of chronotherapeutic approaches to better target endogenous circadian rhythms.

## Results

**Figure 1.**
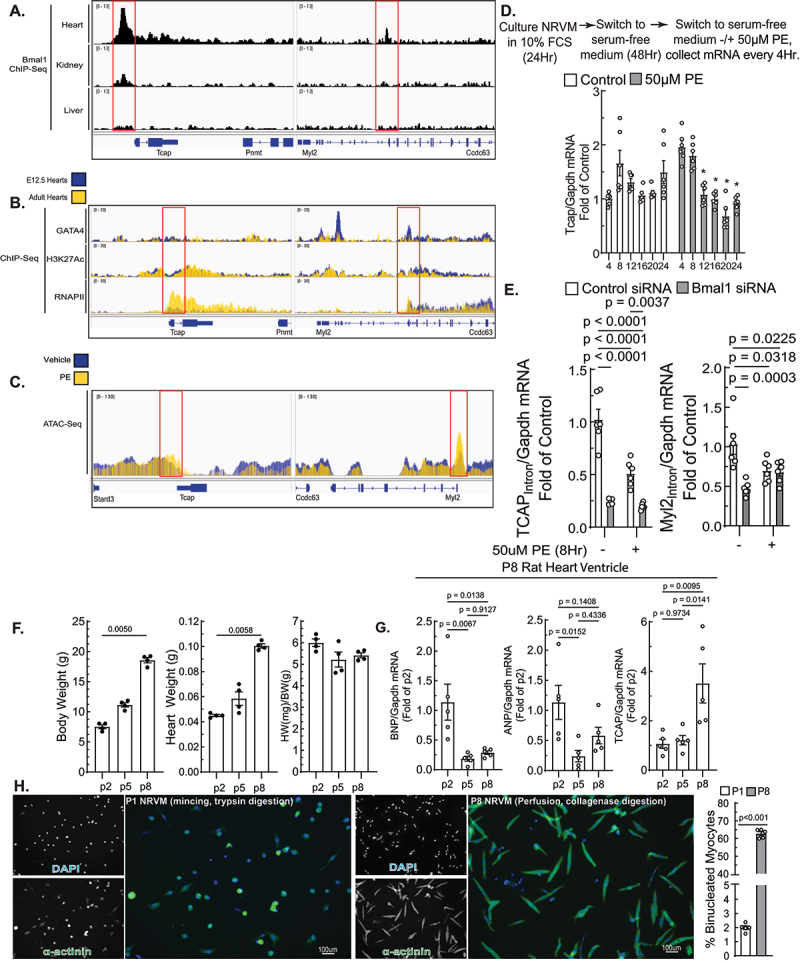
Postnatal and circadian regulation of Tcap and Myl2 expression.

We examined a transcriptomic study of embryonic and adult mouse myocytes and cross-referenced genes significantly upregulated in the adult heart with experimentally determined regions of Bmal1 binding in whole heart, kidney, and tissue as measured by ChIP-seq [4,10,11]. These analyses revealed enrichment of Bmal1 gene targets involved in muscle cell differentiation and development, with Tcap identified as the gene most significantly enriched in Bmal1 binding in the heart as compared to kidney or liver. We also identified Myl2/Mlc2v, another sarcomeric protein expressed early in cardiogenesis and known to mediate calcium sensitivity of sarcomere force generation [12], as a putative clock-controlled gene (Figure 1(A)). We previously reported that depletion of Bmal1 or histone H3.3a, either of which blocks hypertrophy upon α-adrenergic stimulation with phenylephrine (PE) and impairs Tcap transcription [11], indicating a connection between clock-controlled histone turnover, transcriptional remodeling of this locus, and sarcomeric remodeling. While Tcap shows increased RNAPII occupancy in adult vs. embryonic mouse hearts (Figure 1(B)) and increased accessibility in NRVM treated with PE (Figure 1(C)), treatment of NRVM with PE suppressed Tcap mRNA levels (Figure 1(D)). RNAPII occupancy of the Myl2 promoter is largely unchanged in adult vs. embryonic hearts (Figure 1(B)), but similar to TCAP, Myl2 accessibility increases in NRVM with PE treatment (Figure 1(C)). Importantly, like Tcap_intron_ transcription, Myl2_intron_ transcription in NRVM is impaired by Bmal1 depletion and is suppressed in response to acute PE treatment (Figure 1(E), *note: intron-targeted qPCR measures non-spliced mRNA, indicative of de novo transcription*). Together, these results support the concept of circadian control of the sarcomere at the level of Tcap and Myl2 gene transcription.

(A) Bmal1 ChIP-Seq data [4] generated from whole heart, kidney, and liver, emphasizing Titin-Cap and Myl2 cardiac-specific clock-controlled genes. (B) GATA4, H3K27Ac, and RNAPII ChIP-Seq [10] in embryonic vs. adult myocytes. (C) ATAC-Seq analysis of the TCAP and Myl2 genomic loci in NRVM treated with PE for 24 Hr[30]. (D) Time course of NRVM treated with or without PE treatment, followed by measurement of TCAP mRNA levels via RT-qPCR. * indicates *p* < 0.05 from all other timepoints *in the same condition*, via ordinary one-way ANOVA with Tukey’s *post hoc* analysis for pairwise comparisons. (E) NRVM transfected with control or Bmal1-targeting siRNA, with or without PE treatment, followed by measurement of TCAP [11] and Myl2 mRNA levels via RT-qPCR. *p*-values generated via two-way ANOVA with Tukey’s *post hoc* analysis for pairwise comparisons. (F) Heart weight, body weight, heart weight/body weight ratio of neonatal rats at postnatal days 2, 5, and 8. (G) Expression of BNP, ANP, and TCAP across time-of-day-matched animals at postnatal days 2, 5, and 8. Indicated *p*-values generated by ordinary one-way ANOVA with Tukey’s *post hoc* analysis for pairwise comparisons. (H) α-actinin immunocytofluorescence of myocytes isolated from P1 rat hearts via mincing and percoll purification vs. P8 via Langendorff digestion and quantification of binucleation in P1 vs. P8 neonatal rat ventricular myocytes. Indicated *p*-value generated by unpaired t-test with Welch’s correction.

During the postnatal period, the mammalian heart matures in part by myocyte hypertrophy and transcriptional upregulation and incorporation of adult isoforms of sarcomeric proteins during sarcomere remodeling and expansion [13–15]. Because RNAPII occupancy at the Tcap locus increases in the adult vs. embryonic heart, which is not observed with Myl2, we hypothesized that Tcap transcription may be similarly regulated postnatally. We measured body weight, heart weight, fetal gene expression and calculated heart weight/body weight across time-of-day matched neonatal rats ages P2, P5, and P8. Heart weight and body weight were significantly increased between P5 and P8, with no difference in heart weight/bodyweight ratio (Figure 1(F)), indicative of normal physiological growth. P5 and P8 hearts displayed overall suppression of fetal genes Nppa and Nppb, whereas Tcap mRNA levels increased at P8 (Figure 1(G)), a timepoint demarcating the close of the regenerative window and the peak of myocyte binucleation (Figure 1(H)).

**Figure 2.**
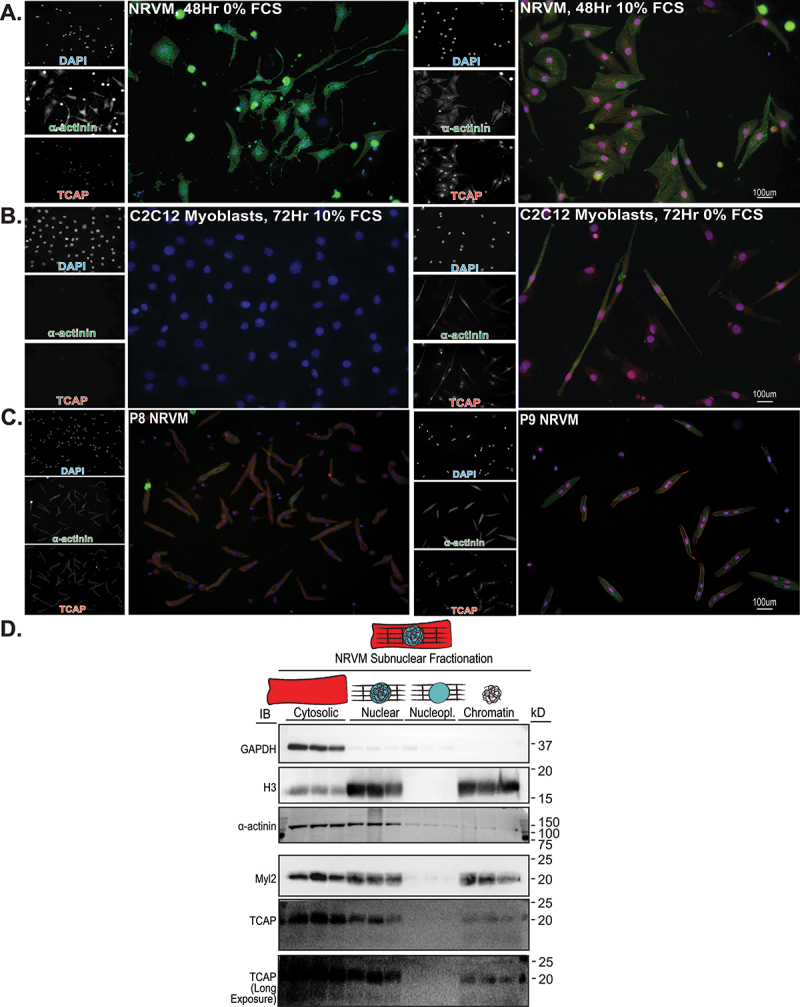
TCAP and Myl2 partially localize to the chromatin compartment of the nucleus.

To visualize TCAP localization in relation to the sarcomere, NRVM maintained in 0% or 10% FCS were subjected to TCAP and α-actinin immunocytofluorescence. NRVM maintained in 10% FCS revealed TCAP immunofluorescence indicative of sarcomeric localization; however, there was clear TCAP nuclear localization independent of serum concentration (Figure 2(A)). Since TCAP is also expressed in skeletal muscle [16], C2C12 skeletal myoblast cultures maintained in 10% FCS (pro-proliferative) or 0% FCS (pro-differentiation) [17] were similarly stained for α‑actinin and TCAP. Intriguingly, under 0% FCS, α-actinin and TCAP co-staining was observed in a fraction of cells visualized, while other cells displayed nuclear TCAP localization in the absence of α-actinin staining (Figure 2(B)). TCAP was reported to interact with P53 in the nucleus [5] and Myl2 exerts gene‑regulatory functions during I/R injury [18]. Accordingly, to assess whether TCAP mobilizes to the nucleus following closure of the postnatal cardiac maturation window, myocytes were isolated from P8 and P9 rat hearts, detecting nuclear localization of TCAP at P9 but not P8 (Figure 2(C)). Finally, we assessed subnuclear localization of these two proteins in NRVM, revealing the presence of both TCAP and MYL2 in the chromatin fraction containing histone H3 (Figure 2(D)).

(A) NRVMs were incubated in 10% FCS for 48 h, followed by α-actinin and TCAP immunocytofluorescence or subnuclear fractionation. (B) As further confirmation of nuclear localization of TCAP, C2C12 myoblasts maintained in 10% or 0% FCS to signal proliferation or differentiation, respectively, where TCAP fluorescence is observed with 0% FCS. (C) Isolation of myocytes from P8 and P9 rat hearts reveal nuclear localization of TCAP at the close of the cardiac maturation window. (D) Subcellular and subnuclear fractions were validated by histone H3, GAPDH, and α-actinin immunoblotting, followed by immunoblots of Myl2 and TCAP.

**Figure 3.**
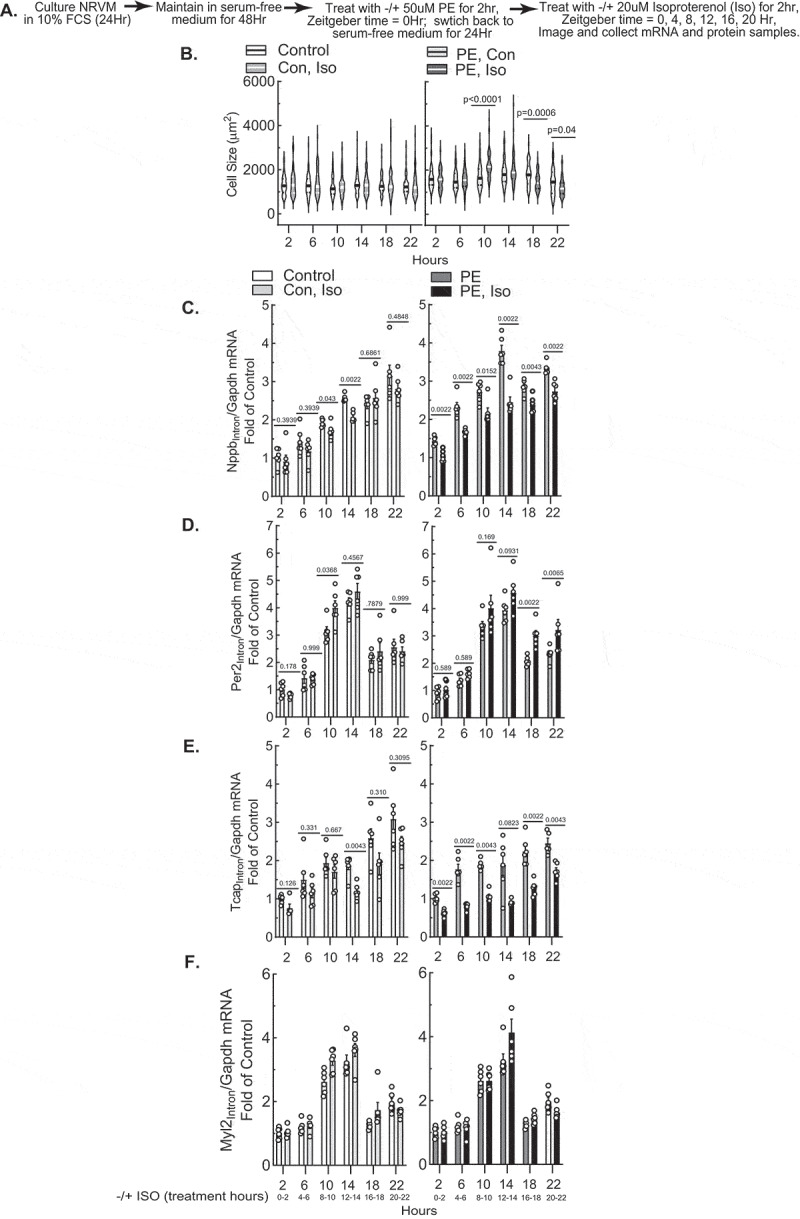
An isoproterenol phase response curve on NRVM treated with phenylephrine reveals effects of β-adrenergic stimulation on myocyte hypertrophy/atrophy and transcription of clock-controlled genes.

Having characterized the normal expression and localization of Tcap in relation to key markers of myocyte maturation under basal conditions, and demonstrated that Myl2 transcription is Bmal1-dependent, we next sought to examine the effects of external cues from catecholamines on TCAP and Myl2 expression during myocyte hypertrophy. α-adrenergic stimulation is sufficient to remodel cardiac-specific clock gene transcription, but the effects of acute β-adrenergic stimulation on cardiac-specific clock gene expression during hypertrophy entrained by α-adrenergic stimulation are unknown. Furthermore, these hormonal pathways do not act in isolation *in vivo*. We therefore administered isoproterenol (ISO) to vehicle- or PE-treated NRVMs and plotted the phase response curves for cell size, transcription of Nppb_intron_, Per2_intron_, Myl2_intron_, Tcap_intron_, and TCAP and MYL2 protein levels (Figure 3(A)). ISO alone did not impact cell size over the studied time span, whereas PE alone induced hypertrophy that peaked at 10 hr and then waned toward the end of the experiment (Figure 3(B)). The phase response curve of ISO treatment following PE treatment revealed time-of-day-dependent myocyte hypertrophy (at ZT10) followed by atrophy (at ZT18, 22) as indicated by cell size measurements (Figure 3(B)). ISO alone attenuated Nppb_intron_ transcription, and this effect was significantly enhanced by PE (Figure 3(C)), whereas Per2_intron_ transcription, which oscillates, was largely impervious to ISO treatment in both vehicle- and PE-treated NRVM (Figure 3(D)). Intriguingly, Tcap_intron_ transcription did not oscillate in the absence or presence of PE, while PE entrenched ISO-mediated suppression of Tcap_intron_ transcription similarly to Nppb_intron_ (Figure 3(E)). Similarly to Per2_intron_, Myl2_intron_ transcription oscillates and is impervious to α- or β- adrenergic stimulation (Figure 3(F)), supporting the notion that Myl2 is a clock-controlled gene.

(A) Experimental workflow of NRVM cell culture to generate an ISO phase response curve following PE treatment. (B, C, D, E, F) Cell size, Nppb_Intron_, Per2_Intron_, TCAP_Intron_, and Myl2_Intron_transcript level measurements. Indicated *p*-values generated by student’s unpaired t-test with Welch’s correction.

**Figure 4.**
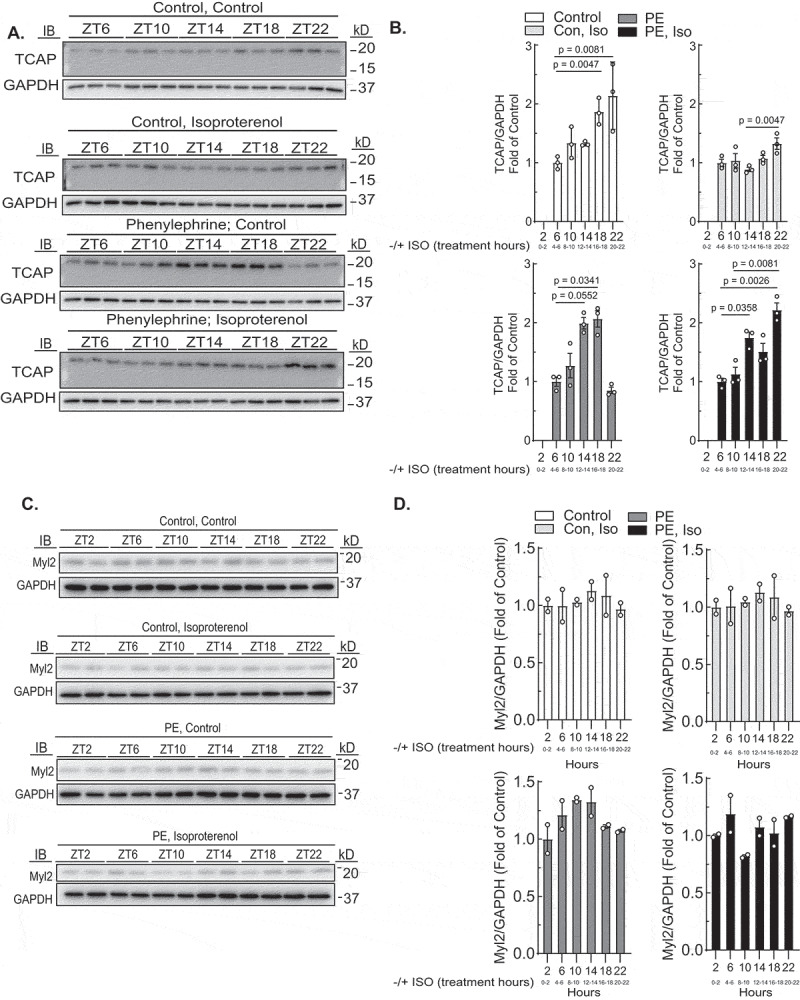
An isoproterenol phase response curve on NRVM treated with phenylephrine reveals effects of β-adrenergic stimulation on TCAP and MYL2 expression. Following the experimental workflow described in Figure 2(A), TCAP, MYL2, and GAPDH immunoblots were performed (A, C) and quantified in (B, D). Indicated *p*-values generated by ordinary one-way ANOVA with Tukey’s *post hoc* analysis for pairwise comparisons.

We observed a time-of-day dependent change in TCAP protein levels after PE (Figure 4(A,B)), in agreement with *de novo* TCAP_intron_ transcription (Figure 3(E)). Combined treatment of ISO and PE did not affect relative levels of TCAP until the final time point measured (ZT22), at which point ISO treatment stimulated TCAP accumulation. Surprisingly, Myl2 expression patterns are inverted from that of TCAP: whereas Myl2_intron_ transcription is time-of-day dependent, Myl2 protein levels did not significantly change across the same period (Figure 4(C,D)). The findings of Figures 3 and 4 establish Myl2 as a novel clock-controlled gene and describe the adrenergic stimulus-induced behavior of Tcap expression. However, myocyte growth and clock-controlled gene expression *in vivo* are strongly impacted by other non-adrenergic stimuli in the microenvironment. Accordingly, we sought to examine this phenomenon by studying the effects of cell-cell communication on myocyte hypertrophy and clock gene expression.

**Figure 5.**
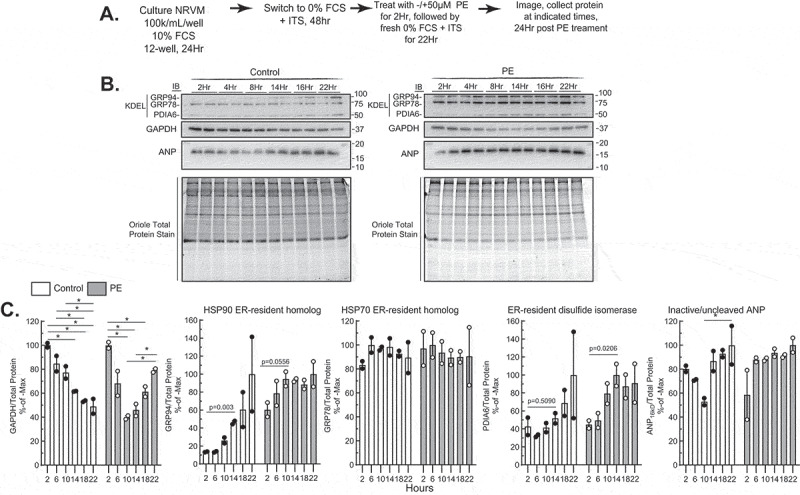
NRVM treated with phenylephrine reveals time-of-day dependent effects of α-adrenergic stimulation on expression of the secreted protein ANP and the ER-resident protein‑folding chaperones GRP94, GRP78, and PDIA6.

Since α-adrenergic stimulation did not result in oscillatory transcription of Tcap_intron_, but did sensitize it to β-adrenergic stimulation, this led us to propose that α-adrenergic stimulation may entrain time-of-day dependent TCAP_intron_ transcription in response to other external variables that influence clock-gene expression. As described above, Figure 1(F,G) demonstrates that heart weight significantly increases between P5 and P8, concurrent with a significant increase in Tcap mRNA levels and suppression of fetal genes consistent with physiological hypertrophy. Previous studies have reported that hypertrophic myocytes secrete paracrine factors that can modulate hypertrophy of other myocytes [19]. In our experiments, whereas vehicle-treated NRVM displayed a slow onset of expression of ER protein‑folding chaperones GRP94 and PDIA6, and time-of-day dependent expression of the ER-trafficked hormone ANP (Figure 5(A,B) (left), 5(C)), PE-treated NRVM showed a faster onset of expression of ER protein‑folding chaperones GRP94 and PDIA6, and ANP levels that were not time-of-day dependent (Figure 5(B), right, 5(C)).

(A) Experimental workflow of NRVM treated with PE treatment. (B) KDEL, GAPDH, ANP immunoblots and total protein oriole fluorescent stain. (C) Quantification of immunoblots/total protein shown in B. Indicated *p*-values generated by ordinary one-way ANOVA with Tukey’s *post hoc* analysis for pairwise comparisons *across the same condition*.

**Figure 6.**
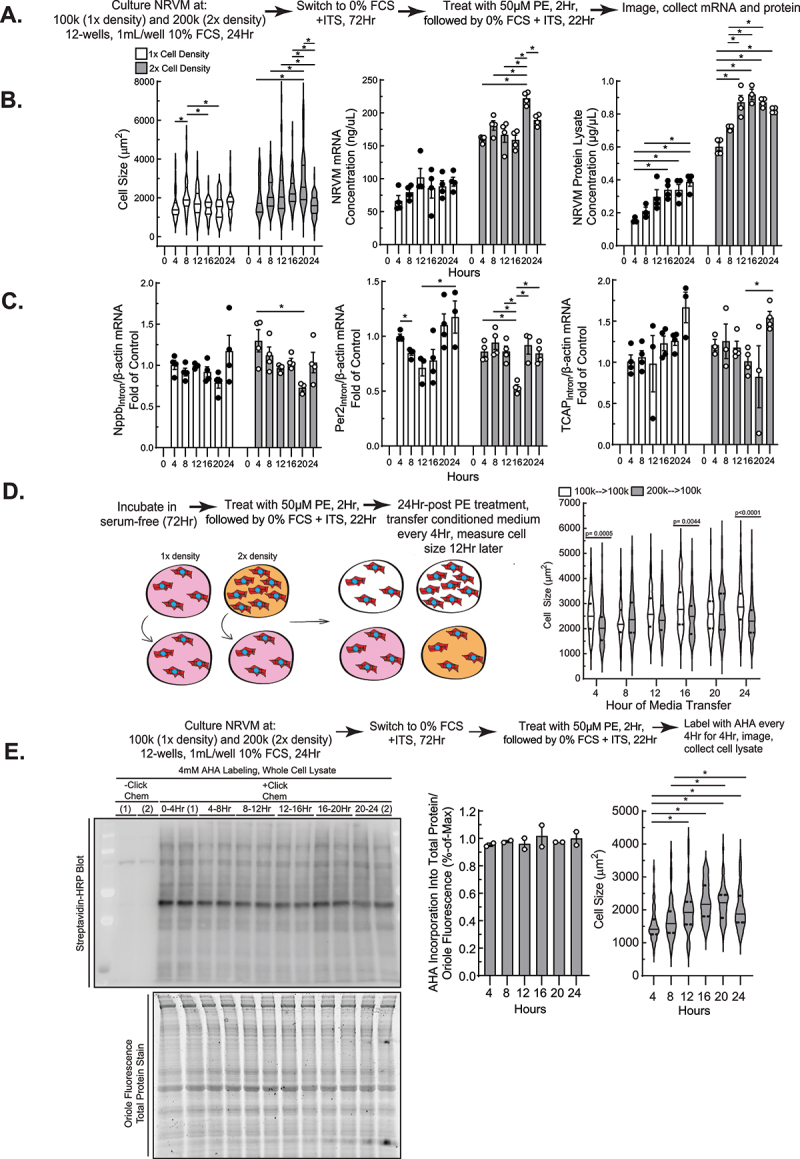
Myocyte biomass influences the kinetics of myocyte growth and Per2 and TCAP transcription.

Since increased myocyte biomass in culture increases the concentration of secreted factors [20], we assessed whether myocyte biomass can influence the kinetics of myocyte hypertrophy and Per2_intron_ and Tcap_intron_ transcription. NRVM were cultured at two different densities (1x vs. 2x), treated with PE, and collected every 4 h, 24 h after PE treatment (Figure 6(A)). Plating cells at a higher density resulted in increased biomass, as reflected by total mRNA and protein measurements. Intriguingly, myocyte size peaked at a later timepoint and with greater amplitude in cells plated at 2x density (Figure 6(B)). Whereas no significant differences were observed in Nppb_intron_ levels at 1x density, at 2x density Nppb_intron_ transcription is suppressed concomitantly with the peak of myocyte size. 1x-density cultures display a biphasic response of suppression followed by induction in Per2_intron_ transcription, which is time‑shifted in the 2x-density cultures. The kinetics of Tcap_intron_ transcription were strongly influenced by cell density, gradually increasing in 1x-density cultures but displaying an abrupt surge between 16 and 24 h in 2x-density cultures (Figure 6(C)). To test the hypothesis that the increase in biomass induces a transferrable environmental change (e.g., a secreted factor) that can alter myocyte growth, the medium of 1x-density cultures was removed and supplanted with 2x-density conditioned medium every 4 hr, starting 24 hr after PE treatment. This was followed by cell‑size measurement 12 h after conditioned medium transfer. Media from 2x-density cells inhibited cell growth when transferred to 1x-density cells (as compared to 1x to 1x transfers) (Figure 6(D)), suggesting a secreted factor that signals to slow cell growth at higher density. Finally, labeling of 2x cultures with the translationally incorporated methionine analog AHA [11,21] did not reveal significant changes in translation across time despite differences in myocyte cell size over a 24-h period (Figure 6(E)), suggesting the increase in cell mass and protein abundance contributing to myocyte hypertrophy is not directly coupled to hypertrophy on a 24-h time scale.

(A) Experimental workflow of NRVM cell culture experiments. (B) Measurement of myocyte cell size, total mRNA, and total protein. (C) Measurement of Nppb_Intron_, Per2_Intron_, and TCAP_Intron_transcription. Indicated *p*-values generated by ordinary one-way ANOVA with Tukey’s *post hoc*analysis for pairwise comparisons. (D) Diagram of conditioned‑medium experiments between cells plated at ‘1x’ and ‘2x’ densities to assess how 2x-conditioned medium affects myocyte size. Indicated *p*-values generated by student’s unpaired t-test with Welch’s correction. (E) After labeling myocytes with 4 mM AHA for 4 h across a 24 h timescale, protein lysates containing newly synthesized AHA-labeled proteins are alkylated via click chemistry with alkyne-biotin and visualized with streptavidin-HRP enhanced chemiluminescence, alongside cell size measurements. * Indicates p<0.05 via ordinary one-way ANOVA with *Tukey’s post hoc analysis* for pairwise comparisons.

**Figure 7.**
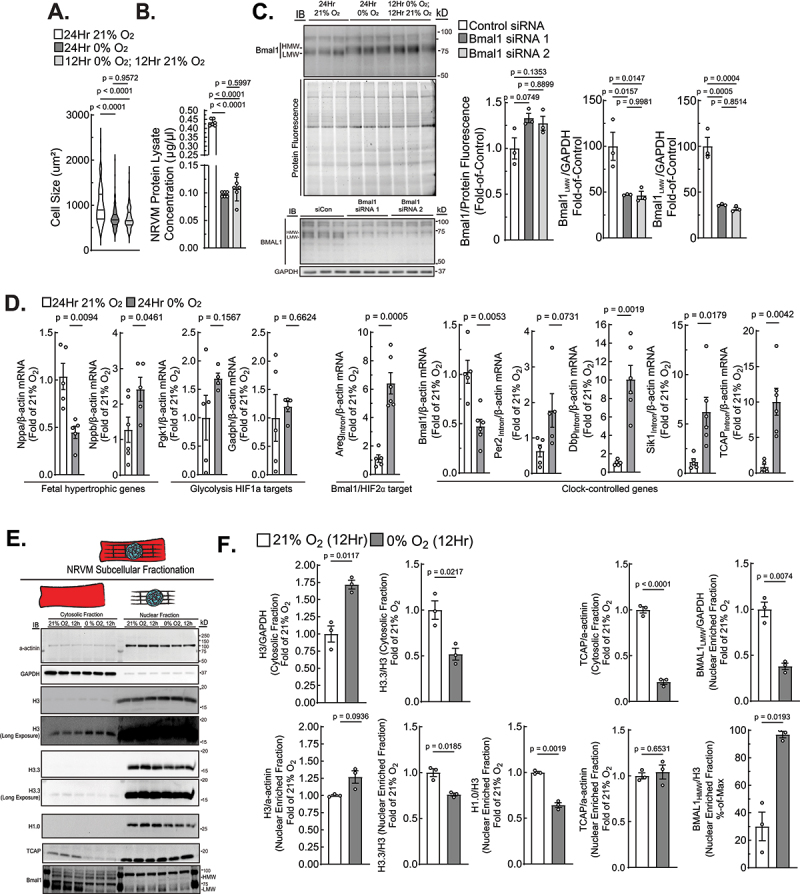
Decreased oxygen causes myocyte atrophy in parallel with changes in clock-controlled gene expression and histone stoichiometry.

Birth is associated with increased O_2_ tension, which sets the timing of closure of the postnatal cardiac regenerative window [14,15,22], shifting from proliferative to hypertrophic growth. We reasoned that control of cardiac myocyte circadian gene expression may be influenced by O_2_ tension as part of the cellular mechanism for myocyte maturation and cell cycle exit. To test this hypothesis, NRVM were subjected to 0% O_2_ for 24 h, or 0% O_2_ for 12 h followed by 12 Hr 21% O_2_, both of which significantly decreased cell size and total protein as compared to control conditions (Figure 7(A,B)). Our previous work had revealed transient changes in histone stoichiometry associated with myocyte growth [11], so we examined this endpoint alongside Bmal1 levels under decreased O_2_ tension. Bmal1 immunoblots revealed overall no changes in Bmal1 levels with 0% O_2_ with or without reoxygenation but showed an upward shift in Bmal1 molecular weight with exposure to 0% O_2_. NRVM transfected with two different siRNAs targeting Bmal1 revealed a significant decrease in both Bmal1 protein products (Figure 7(C)). 0% O_2_ suppressed Nppa and increased Nppb transcript levels (Figure 7(D)). To confirm that myocyte cultures were experiencing and responding to decreased O_2_ tension, transcript levels of HIF-1α targets [23,24] Pgk-1 and Gapdh and HIF2α/Bmal1 target [25] amphiregulin (Areg) were assessed. Consistent with previous work reporting that HIF-1α transcriptional activity is optimal at 1% O_2_ but compromised at 0% O_2_^24^, 0% O_2_ did not induce Gapdh or Pgk1. However, 0% O_2_ did induce Areg_intron_ transcription (Figure 7D), suggesting that HIF2α induces target gene expression at O_2_ levels at which HIF1α is inactive. To investigate whether these changes in cell phenotype operate by reprogramming the molecular clock, we measured transcription of the core clock genes Per2_intron_ and DBP_intron_ (negative clock regulators), and the cardiac-specific clock target genes Sik1_intron_ and Tcap_intron_, all of which were upregulated, whereas Bmal1 (positive clock regulator) was suppressed (Figure 7(D)).

(A) Cell size and (B) total protein measurements of myocytes subjected to 24 h of 0% O_2_ or 12 Hr 0% O_2_followed by 12 Hr 21% O_2_. (C) Immunoblots and quantifications of NRVM whole cell lysate for Bmal1 alongside total protein via oriole fluorescence staining, or GAPDH, following 0% O_2_treatment or transfection with Bmal1-targeting siRNA. Indicated *p*-values generated via ordinary one-way ANOVA with Tukey’s*post hoc*analysis for pairwise comparisons. (D) RT-qPCR for indicated fetal hypertrophic, glycolytic, or clock-controlled genes in NRVM subjected to 21% or 0% O_2_. Indicated *p*-values generated by student’s unpaired t-test with Welch’s correction. (E, F) Subcellular fractionation of NRVM subjected to 21% or 0% O_2_ treatment for 12 h, followed by immunoblots of α-actinin, GAPDH, H3, H3.3, H1.0, TCAP, and Bmal1. Indicated *p*-values generated by student’s unpaired t-test with Welch’s correction.

Subcellular fractionation of myocytes following 12 h of 0% O_2_ revealed cytosolic accumulation of histone H3 and decreased levels of chromatin-associated histone H3.3 and H1.0 (histone variants that undergo turnover independent of DNA replication [21,26]). Additionally, these effects occur concomitantly with decreased cytosolic Bmal1 and increased high‑molecular‑weight BMAL1 in the nuclear-enriched fraction—consistent with a previous report of high-molecular‑weight BMAL1 [27]. Cytosolic TCAP levels decrease similarly to BMAL1; however, TCAP levels did not change in the nuclei-enriched fraction (Figure 7(E,F)). These results indicate that decreased O_2_ tension suppresses myocyte growth, induces negative regulators of the circadian clock, induces Tcap_intron_ transcription, alters chromatin-associated histone stoichiometry, and alters the distribution of TCAP and BMAL1 between the cytosolic and nuclear compartments.

**Figure 8.**
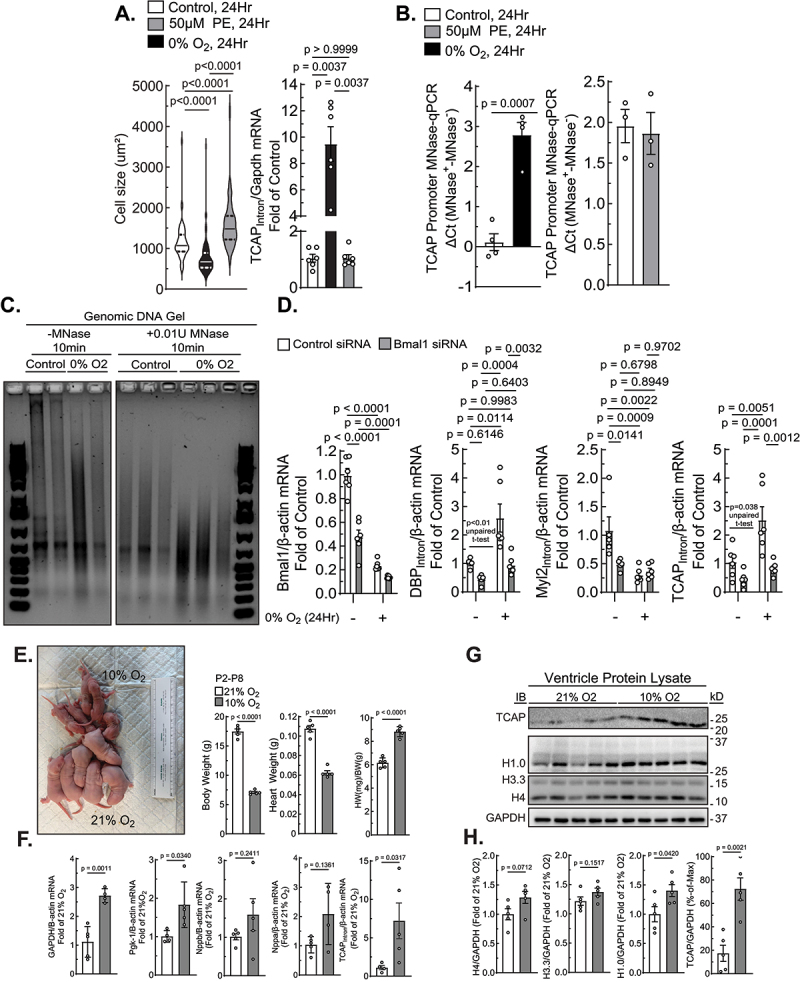
The circadian clock transcriptionally remodels Tcap expression during hypoxia-driven changes in chromatin organization.

Since 0% O_2_ altered chromatin-associated histone stoichiometry, while also driving TCAP induction, we assessed whether 0% O_2_ altered global chromatin accessibility, accessibility of the TCAP locus, and whether Bmal1 is required for 0% O_2_-mediated induction of Tcap. 24 hours of 0% O_2_ decreased cell size, whereas 50 µM PE increased cell size, concomitant with induction of TCAP_intron_ transcription by 0% O_2_ but no effect with PE treatment (Figure 8(A)). In agreement with this effect on transcription, 0% O_2_ but not PE increased TCAP promoter accessibility as assessed by MNase-qPCR (Figure 8(B); 0% O_2_ also increased global chromatin accessibility Figure 8(C)). Depletion of Bmal1 attenuated 0% O_2_-mediated induction of TCAP_intron_ and DBP_intron_, whereas the effect of 0% O_2_ to suppress Myl2_intron_. transcription was independent of Bmal1 (Figure 8(D)).

A) Cell size and TCAP_intron_RT-qPCR measurements of myocytes subjected to 24 h of 0% O_2_ or 50uM PE. B) Changes in TCAP promoter accessibility (indicative of MNase degradation) as measured by MNase-qPCR for the TCAP promoter, highlighted in Figure 1(C), following 24 h of 0% O_2_or 50 µM PE. C) DNA gel following MNase digestion for examination of global chromatin compaction. D) NRVM were subjected to Bmal1 knockdown followed by 0% O_2_. Bmal1 transcript levels were measured by RT-qPCR, while transcription of additional clock-controlled gene levels measured by RT-qPCR for DBP_Intron_, Myl2_Intron_, and Tcap_Intron_, (indicated *p*-values generated by two-way ANOVA with Tukey’s post hoc analysis for pairwise comparisons). E) Image of neonatal rat litters subjected to 21% vs. 10% O_2_ from P2-P8, and measurements of heart weight, body weight, and heart weight/body weight. F) RT-qPCR of fetal hypertrophic genes, glycolytic genes, and TCAP_Intron_. G, H) Immunoblots of ventricular tissue for TCAP, H1.0, H3.3, H4, and GAPDH, along with quantifications. Indicated *p*-values generated by student’s unpaired t-test with Welch’s correction.

We next determined whether *in vivo* hypoxia would drive similar changes in histone levels and Tcap_intron_ transcription by subjecting neonatal rat litters to 21% (normoxia) versus 10% (hypoxia) O_2_ from P2-P8. Hypoxic animals displayed significantly decreased HW and BW, but increased HW/BW ratio (Figure 8(E)). In contrast to NRVM subjected to 0% O_2_, hearts of animals subjected to 10% O_2_ displayed increased expression of Gapdh and Pgk1, while Nppa and Nppb transcript levels were unchanged (Figure 8(F)). Consistent with what was observed in NRVM, TCAP_intron_ transcription was significantly increased in hypoxic hearts (Figure 8(F)), while immunoblotting revealed increased histone H1.0 levels, unchanged histone H3.3 and H4 levels, and increased TCAP levels (Figure 8(G,H)).

**Figure 9.**
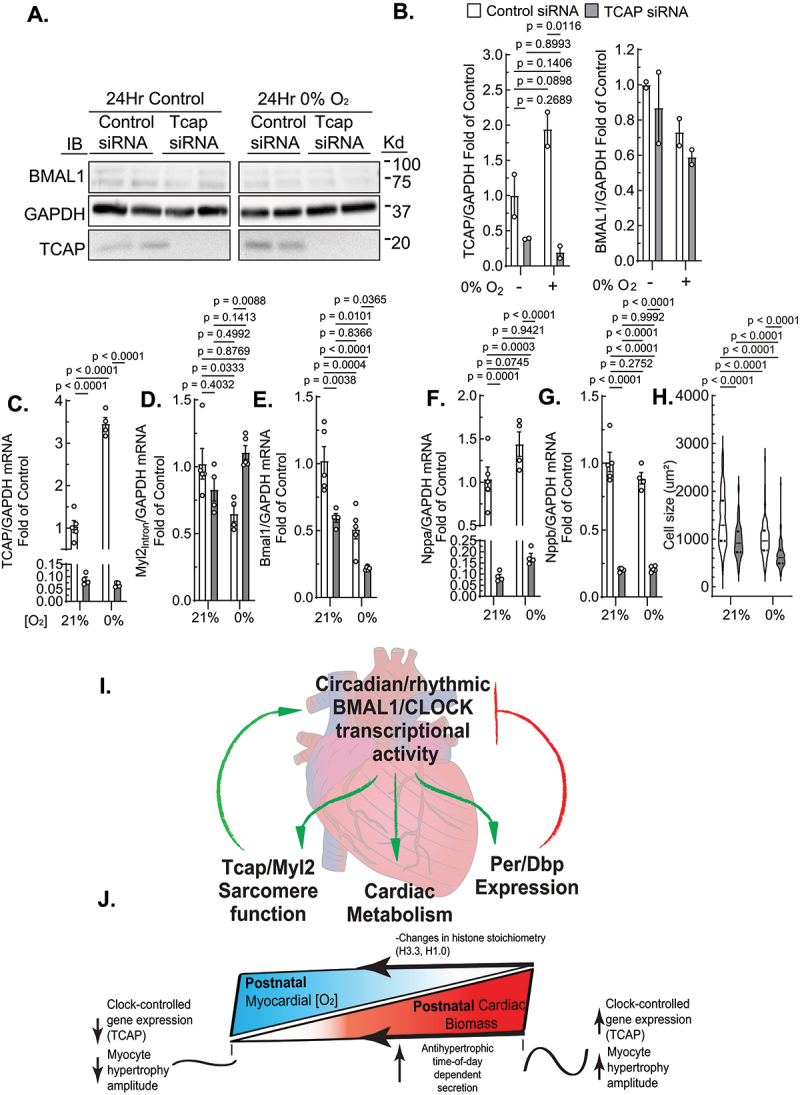
TCAP depletion impairs expression of clock-controlled and hypertrophic genes. NRVM were transfected with control or TCAP-targeted siRNA followed by subjection to 0 % O_2_ for 24 h. Cultures were imaged for cell‑size measurements, followed by collection of whole‑cell protein lysate and total RNA.

Since TCAP is induced in response to decreased O_2_ tension observed in both our *in vitro* and *in vivo* experiments, we assessed how TCAP depletion affects myocyte atrophy in response to 0% O_2_ and the expression of fetal hypertrophic and clock-controlled genes. Anoxia decreased cell size and Bmal1 expression levels while increasing TCAP expression (Figure 9(A-C)). Whereas Bmal1 depletion had no effect on 0% O_2_-mediated suppression of Myl2_intron_ transcription (Figure 8(D)), TCAP depletion was sufficient to regulate Myl2_intron_ transcription (Figure 9(D)). Furthermore, TCAP depletion alone suppressed Bmal1 and fetal gene mRNA levels (Figure 9(E-G)) and when coupled with 0% O_2_ resulted in a 78% decrease in Bmal1 mRNA levels. No further effect was observed on fetal hypertrophic gene expression when TCAP depletion was coupled with 0% O_2_ (Figure 9(F,G)), whereas TCAP depletion and 0% O_2_ additively decreased myocyte size (Figure 9(H)).

(A, B) TCAP, BMAL1, and GAPDH immunoblots and their quantification. (C-G) TCAP_intron_, Myl2_intron_, Bmal1, Nppa, and Nppb mRNA levels as measured by RT-qPCR, and (H) cell size measurements as measured by microscopy. *p*-values indicated generated by two-way ANOVA followed by Tukey’s post hoc analysis. (I, J) Summary conceptual diagram of the interpretation of results presented in this study. (I) The circadian clock, through Bmal1/CLOCK influence cardiac-specific processes, while Bmal1/CLOCK induces negative clock regulators. Here, the sarcomere, via TCAP and Myl2, positively feeds back onto the core clock mechanism. (J) As the heart accumulates biomass postnatally and undergoes changes in O_2_ tension, histone stoichiometry is altered and TCAP is induced and/or becomes more oscillatory in its function(s) to continue supporting postnatal cardiac maturation and hypertrophy.

## Discussion

In this study, we set out to determine the mechanisms by which the circadian clock exerts control over TCAP and Myl2 expression, as these genes represent key regulators of sarcomere function [5,8,12] and thus myocyte maturation. α-adrenergic stimulation entrained time-of-day dependent myocyte hypertrophy and atrophy, as well as transcriptional suppression of Tcap_intron_ and Nppb_intron_ following subsequent β-adrenergic stimulation. In contrast, oscillatory Myl2_intron_ and Per2_intron_ transcription were largely impervious to β-adrenergic stimulation. TCAP protein oscillation is enforced by α-adrenergic stimulation, temporally sensitive to β-adrenergic stimulation, and independent of *de novo* Tcap transcription—properties not demonstrated by MYL2. Our results also indicate that myocyte biomass, likely via paracrine and autocrine signaling, influences timing and amplitude of myocyte hypertrophy and clock-controlled gene transcription, independent of global changes in translation. Additionally, decreased O_2_ tension results in decreased levels of chromatin-associated histone H3.3 and H1.0, while driving increased TCAP promoter accessibility and Bmal1-dependent transcription. Finally, we demonstrate both TCAP and Myl2 localize to the chromatin subnuclear fraction of NRVM and that, as a cardiomyocyte core clock gene, TCAP influences expression of both Bmal1 and Myl2: this observation raises the question of whether MYL2 and TCAP influence chromatin beyond modulating expression of individual genes [18], or activity of individual transcription factors in response to non-mechanical cues [5].

TCAP protein oscillations in the heart have previously been reported [3]. However, this is the first report dissecting the external cues controlling this process and documenting the mobilization of TCAP to the nucleus in the context of muscle cell differentiation (Figure 6(B)) and postnatal cardiac maturation (Figure 6(C)). Previous studies in skeletal muscle have implicated the clock-controlled ubiquitin ligase Fbxl21 in TCAP degradation [28]. However, in our analysis of the relevant Bmal1 ChIP-Seq dataset [4] we did not identify this gene as a clock-controlled target in the heart. Here, PE stimulates TCAP protein oscillation independently of Tcap transcription and entrenches time-of-day dependent TCAP protein accumulation in response to ISO. MYL2 was also detected in the chromatin compartment alongside TCAP, but in contrast to TCAP, MYL2 protein levels did not change in response to α- or β-adrenergic stimulation, despite time-dependent changes in Myl2 gene transcription. These results highlight an interconnection between α- and β- adrenergic signaling mechanisms controlling protein expression independent of the transcriptional states of TCAP and MYL2. Further investigation is required to determine: the totality of TCAP and MYL2 chromatin‑compartment interactomes, whether chromatin-associated MYL2 and TCAP levels are under circadian control, and what role these proteins play in perinatal cardiac development. Additionally, the phase‑response curve of β-adrenergic stimulation following α-adrenergic treatment indicates that α-adrenergic stimulation can alter the transcriptional response of one clock-controlled gene (Tcap) to a different stimulus, without affecting the ongoing transcription of others (Per2, Myl2), all in the absence of the initial α-adrenergic agonist. These results raise the question of how these different responses are entrained at the chromatin level, how the circadian clock resolves their response upon stimulation with the endogenous α/β-adrenergic agonist catecholamine norepinephrine, and whether other hypertrophic GPCR agonists such as ET-1 [29] and AngII [19] influence cardiac-specific clock-controlled gene expression.

Transcription of Tcap and Myl2 was predicted to oscillate independently of external cues, similar to what we observed for the established clock gene Per2: our data indicate that transcription kinetics of Myl2 were similar to that of Per2, which was not observed with Tcap. The phase response curves plotted in (Figures 7(D,F)) indicate that Per2 and Myl2 transcription is insensitive to α- or β- adrenergic stimulation. Furthermore, differences in Per2 and Tcap transcription can be observed in myocyte cultures plated at different densities, concomitant with differences in the timing and amplitude of myocyte hypertrophy (Figure 6(A-C)). These results, coupled with the observation that conditioned medium of cells plated at higher densities can alter the growth dynamics of cultures plated at lower densities (Figure 6(D)) suggest that secreted factors, the levels of which are directly influenced by myocyte number and biomass, influence the timing of clock-controlled gene transcription. This notion is further supported by the observation that Tcap mRNA increases at postnatal day 8, when heart mass significantly increases and the cardiomyocyte proliferative window has closed, as indicated by binucleation (Figure 5(H)). Future studies are needed to identify the secreted factor(s) exerting influence over both myocyte growth and clock-controlled gene expression, and whether TCAP influences chromatin organization, including that of genes encoding secreted factors, such as Nppa and Nppb.

While our data suggest that myocyte biomass and cell communication may influence the expression of Tcap, they also suggest Tcap transcription is under O_2_-sensing control mechanisms. In hypoxic neonatal hearts, Tcap transcriptional induction co-occurs with increased expression of hypoxia-inducible targets (Figure 8(E-H)), and we demonstrate in isolated cells subjected to anoxia that induction of TCAP is Bmal1-dependent (Figure 8(D)). It has been recently demonstrated that Bmal1 heterodimerizes with HIF2α and transcriptionally induces non-canonical clock-controlled gene targets under hypoxic conditions [25]. Under anoxic conditions in culture, we observe increased transcription of one of these targets, Areg, suggestive of a potential mechanism warranting future investigation: that is, Bmal1/HIF2α coupling of cardiac contractility to O_2_‑sensing via transcriptional regulation of Tcap. In our previous study [11], we demonstrated that depletion of histone H3.3 or Bmal1 impaired transcription of clock-controlled genes. In the present study, we also demonstrate that decreased O_2_ decreases chromatin-associated histone H3.3 and H1.0 levels, concomitant with accumulation of high-MW BMAL1 in a nuclei-enriched fraction (Figure 7(E,F)), a global decrease in chromatin compaction, and increased accessibility, of the Tcap promoter (Figure 8(B-D)). As TCAP is induced with decreased O_2_ tension, localizes to the nucleus, and influences clock gene expression, our results suggest that TCAP exerts O_2_-sensitive functions to dictate chromatin organization and function in cardiac myocytes, a notion that will require further experimentation including interrogation of global TCAP genome occupancy.

A previous study reported that the TCAP promoter can drive luciferase reporter activity in a BMAL1/CLOCK-dependent manner; coupled with the observation that TCAP protein levels oscillate as measured in whole-heart lysate, the conclusions drawn were that the circadian clock may influence sarcomere function via TCAP as a circadian clock output gene [3]. A prior study suggested that, in response to biomechanical stress, TCAP traffics to the nucleus to attenuate cell‑death transcription programs [5]. Our experiments demonstrating that TCAP depletion is sufficient to impair basal Bmal1 expression and transcriptional remodeling of Myl2 in response to 0% O_2_ (Figure 9(D,E)) suggest that TCAP acts as a core clock gene in cardiomyocytes and influences the sarcomere via transcriptional regulation of Myl2. Further experimentation will be needed to determine if TCAP localization to the chromatin compartment during postnatal cardiomyocyte maturation specifically influences Myl2 transcriptional remodeling and ensuing sarcomere assembly processes.

In summary, we present evidence that the circadian clock regulates Myl2 and Tcap expression in neonatal ventricular myocytes, and that Tcap regulation as a myocyte core clock gene is influenced by multiple external cues. Previous studies have focused on the influence of catecholamines and glucocorticoids on clock-controlled gene expression. Here we provide evidence for cues coming from other myocytes, and for the role of O_2_ tension in transcriptional remodeling of clock-controlled genes, that support the following concepts (Figure 9(I,J)): Postnatal cardiac growth rapidly consumes oxygen, decreasing myocardial O_2_ tension and thereby limiting heart growth. This hypoxic break on growth induces negative clock regulators to slow down clock-controlled O_2_-dependent processes and induces TCAP to increase sarcomere tension and function and cardiac contractility. Concurrently, antihypertrophic secreted factors, the quantity and composition of which can also be influenced by changes in O_2_ tension and myocyte biomass, serve as a rheostat of postnatal growth to preserve myocardial O_2_ tension.

Future studies are warranted to understand how myocyte paracrine and autocrine signaling, in conjunction with changes in O_2_ tension and MYL2/TCAP chromatinization, entrench clock-controlled gene transcription to drive myocyte growth dynamics. The findings presented here are of critical relevance to perinatal care: improved understanding of the circadian influence of environmental cues to drive postnatal myocardial growth, and subsequent development of chronotherapeutics to deploy in the neonatal intensive care unit, may improve the standard of care and cardiovascular outcomes for prematurely born infants.

## Experimental procedures

All animal studies were approved by the UCLA Animal Research Committee (ARC) in compliance with the National Institutes of Health Guide for the Care and Use of Laboratory Animals.

### Biological replicates in statistical reporting

In this study, all individual data points represent biological replicates. For *in vitro* experiments, each data point represents a separate culture well, and *in vivo*, each replicate represents an individual animal.

### IGV browser track viewing

The BWfiles [4,10] or bedgraph files [30] shown in Figure 5 were downloaded from NCBI gene expression omnibus and displayed on Integrated Genomics Viewer (IGV).

### Isolation of NRVMs from rat hearts

Four litters of neonatal pups (approximately 48 animals, P3) were euthanized by decapitation, and atria were removed from excised hearts. Ventricles were then briefly rinsed in ice-cold 1× ADS buffer (116 mM NaCl, 18 mM Hepes, 845 mM NaHPO_4_, 5.55 mM glucose, 5.37 mM KCl, 831 mM MgSO_4_, and 0.002% phenol red, pH 7.35 ± 0.5) and minced via 200 cuts with sterile micro scissors. Minced ventricles were then subjected to 5 to 10 serial digestions in a Wheaton 356945 Celstir 50 ml Jacketed Glass Spinner Flask with Double Sidearms at room temperature (21 C) using 300 ml/heart of ADS buffer containing 0.1% Trypsin (Sigma-Aldrich, Cat. No. T4799) and 0.002% DNase (Worthington Biochemical Corp, Cat. No. LS002006). Each digestion lasted for 10 to 20 min. The cell suspension from the first digestion was discarded as it contained unwanted dead cells and red blood cells. The cell suspension from each subsequent digestion was then combined with FCS to a concentration of 50% FCS. The pooled cell suspensions were then filtered with a 100 mm nylon cell strainer (Foxx Life Sciences, Cat. No. 410–0003-OEM) and then centrifuged for 8 min at 1500 g. The resulting cell pellet was then resuspended in 8 ml of 1×ADS buffer. Stock Percoll was prepared by combining nine parts of Percoll (cat# 170891-02, GE HealthCare) with one part of clear (without phenol red) 10×ADS. The stock Percoll was used to make the Percoll for the top (density = 1.059 g/ml; 1 part Percoll stock added to 1.2 parts 1× ADS without phenol red) and bottom (density = 1.082 g/ml; 1 part Percoll stock added to 0.54 parts 1× ADS with phenol red) layers. The gradient, consisting of 4 ml top Percoll and 3 ml bottom Percoll, was set in a 15 ml conical tube by pipetting the top Percoll first, and layering the bottom Percoll gently underneath. The cells (in 2 ml red 1× ADS buffer) were layered on the top of the discontinuous Percoll gradient (4 gradients in total) and centrifuged at 1500 g for 30 min at 4 C, with no deceleration break to separate the myocytes from nonmyocytes. The myocytes, concentrated between the two Percoll layers as well as myocytes that centrifuged to the bottom of the tube, were then collected, washed once with 1× ADS buffer, and resuspended in plating medium composed of Dulbecco’s modified Eagle’s medium (DMEM)/F:12 (Thermo Fisher Scientific, Cat. No. 11330-32) supplemented with 10% FCS and penicillin/streptomycin.

### Plating of NRVMs

Fibronectin (Thermo Fisher Scientific, Cat No. 33016015) dissolved at 1 mg/ml in DNase/RNase-free molecular-grade water was further diluted to 5 mg/ml in DMEM/F:12 containing penicillin/streptomycin and then added at 1 ml/well to 6-well dishes, or 0.5 ml/well to 12-well dishes (Corning, Cat. No. 3516, 3513) which were then incubated at 37°C for 30 min. The fibronectin solution was aspirated from the dishes, and the Percoll-purified myocytes suspended in plating medium (10% FCS). In Figure 6, cells were plated on 6-wells plates at approximately 300,000 cells/well/2 mL, representing a 1.5x density. In Figure 7, cells were plated at 100,000 or 200,000 cells/mL/well were then cultured on the fibronectin-treated 12-well dishes, with each well constituting an independent biological replicate. Cultures were then incubated at 37°C in an incubator infused with 95% N_2_/5% CO_2_ for 24 h.

*siRNA sequences*

siRNA oligonucleotides were purchased from integrated DNA technologies (IDT)

Negative Control DsiRNA (5 nmol, Cat. No. 51-01-14-04), a.k.a. ‘scrambled siRNA’ is a nontargeting siRNA indicated by IDT to not interact with sequences in the human, mouse, or rat transcriptomes.

Bmal1 siRNA 1: 5′-rArGrUrArGrArArUrArCrArUrUrGrUrCrUrCrArArCrCrAAC-3′

Bmal1 siRNA 2: 5′-rCrArUrCrCrArArArArGrArUrArUrUrGrCrCrArArArGrUTA-3′

Tcap siRNA 1: 5’-rGrCrArArUrArArArCrGrCrUrCrArGrCrCrArUrGrUrCrCCA-3’

Tcap siRNA 2: 5’-rArArGrArArGrGrArArUrArArUrGrGrCrCrArCrUrUrCrAGA-3’

### Subnuclear fractionation

To isolate nuclei, NRVM cultures (5–6 wells per sample) were washed twice with 1 ml/well ice-cold DPBS and lysed with 100 μl of lysis buffer composed of 10 mM Tris (pH 7.4), 250 mM sucrose, 1 mM EDTA, 0.15% Nonidet P-40 subsitute, and protease/phosphatase inhibitors, and scraped into Eppendorf tubes kept on ice. Samples were then centrifuged at 1000 g for 10 min, yielding a crude nuclear pellet. The crude nuclear pellet was then resuspended in 100 μl and layered on top of a 300 μl sucrose cushion composed on 1.6 M sucrose, 15 mM NaCl, 10 mM Tris pH 7.4, and protease/phosphatase inhibitors. This was followed by centrifugation at 7500 g for 10 min. The resulting nuclear pellet was then washed once with lysis buffer composed of 10 mM Tris (pH 7.4), 250 mM sucrose, 1 mM EDTA, 0.15% Nonidet P-40 subsitute, and protease/phosphatase inhibitors and centrifuged at 7500 g for 5 min. Chromatin was isolated from nuclei by resuspending nuclei in lysis buffer composed of 20 mM Hepes, 7.5 mM MgCl_2_, 30 mM NaCl, 1 M urea, 1% Nonidet P-40 subsitute, and protease/phosphatase inhibitors. This was followed by centrifugation at 13,000 g for 15 min, resulting in a supernatant containing the nucleoplasm and solubilized nuclear membranes and a chromatin pellet. The chromatin pellet was then incubated at 100°C for 10 min in 100 μl of cell lysis buffer composed of 50 mM Tris (pH 8), 1% SDS, 1× protease/phosphatase inhibitor, followed by vigorous vertexing, and centrifugation at 20,000 g for 10 min at 4°C. Protein concentration of each fraction was quantified via bicinchoninic acid assay, and equal percentages of each fraction were loaded and resolved on 15% SDS-PAGE gels followed by immunoblotting as described below.

### MNase-qPCR

Medium was removed from 6-well culture dishes, and adherent cells were washed with ice-cold DPBS and then lysed with 100 μl of ice-cold lysis buffer composed of 10 mM Tris–HCl, pH 7.4, 10 mM NaCl, 2 mM MgCl_2_, 0.5% Nonidet P-40_Substitute_ and 1× protease/phosphatase inhibitor mixture. Lysates/intact nuclei were scraped and transferred to microcentrifuge tubes. For this specific protocol, each sample was composed of one well of a 6-well plate. The resulting crude nuclear suspensions were then centrifuged at 1000 g for 10 min. At this point, the samples were maintained on ice. The supernatant was discarded, and the crude nuclear pellet was then gently but thoroughly resuspended in 120 μl of prewarmed MNase reaction buffer, followed by addition of 120 μl of prewarmed MNase reaction buffer (composed of 10 mM Tris, pH 7.4, 5 mM NaCl, and 1 mM CaCl_2_•2 H_2_O) containing 0 U, 0.001 U, 0.01 U, or 0.1 U MNase (MNase, Worthington, LS004798). The reaction vessels were then immediately placed in a 37°C water bath for 5 min. This was followed by addition of 99 μl of MNase stop reaction buffer composed of 60 μL 100 mM EDTA, 10 mM EGTA, 30 μl 20% SDS, and 9 μl of 25 mg/ml Proteinase K. The resulting solutions were then incubated for 16 h at 37°C. Afterward, 340 μl of phenol:chloroform:isoamyl alcohol (25:24:1) was then added to each tube and gently shaken by hand for 20 s. Samples were then centrifuged at 16,000 g at room temperature for 5 min. The upper aqueous layer was then transferred to a 2 ml Eppendorf tube. To each tube, the following were added in the stated order: 4.25 μl of 15 mg/ml Glycoblue (Thermo Fisher Scientific, Cat. No. AM9515), 170 μl 7.5 M ammonium acetate, and 1.27 ml 100% reagent-grade ethanol. Samples were then stored at − 80°C for 1 h. The samples were then centrifuged at 16,000 g for 30 min at 4°C to pellet DNA. The supernatant was discarded, and 150 μl of 70% reagent-grade ethanol was added, followed by centrifugation at 16,000 g for 2 min at 4°C. The supernatant was discarded, and another 150 μl of 70% reagent-grade ethanol was added, followed by centrifugation at 16,000 g for 2 min at 4°C. The supernatant was discarded, and the DNA pellets were allowed to air‑dry, followed by resuspension in 20 µl DNase/RNase-free water and quantification by nanodrop. Subsequently, 100 ng of DNA samples were resolved via electrophoresis on a 1% agarose gel in 1× Tris acetic acid EDTA buffer, 120 V, 1 h at room temperature (21 °C). Chromatin accessibility was then assessed via RT-qPCR using the following reaction components: 5 µl diluted genomic DNA (50 ng/reaction), 1 µl of a 5 µM of a forward primer stock, 1 µl of a 5 µM reverse primer stock, 3 µl of RNase/DNase‑free water, and 10 µl SsoFast EvaGreen Supermix. Primers were targeted to TCAP promoter highlighted in Figure 5C:

Forward: 5’-CCATCACCACCAGTGAGTCT-3’

Reverse: 5’-CGTCCTCGGAGCCCTTT-3’

### Cell size measurements

NRVM cell size was measured via ImageJ. To accurately measure cell size in µm^2^, an image of a hemocytometer (Thermo Fisher Scientific, Cat. No. 02–671-6) was used to set the scale in ImageJ, and the individual cells were manually traced on a Lenovo T460 Thinkpad with touchscreen.

### mRNA isolation

Medium was removed from 6-well culture dishes, and adherent cells were washed with ice-cold DPBS and then lysed with 300 ml of TRIzol Reagent (Thermo Fisher Scientific, Cat. No. 15,596,026). The resulting lysates were homogenized and scraped into Eppendorf tubes and stored at −80 C. After thawing samples at room temperature (20 C), 60 ml of chloroform was added to each sample and shaken vigorously by hand for 15 s. Samples were then incubated at room temperature for 2 to 3 min to allow separation of layers. The samples were then centrifuged at 13,000 rpm for 15 min at 4 C. The resulting upper clear layer (approximately 150 ml) from each sample was pipetted into a fresh Eppendorf tube, and 150 ml of isopropanol was added to each sample, followed by mixing via 10 inversions. The samples were then centrifuged at 13,000 rpm for 20 min at 4 C. At this point, the samples are kept on ice, and the supernatant was removed without disturbing the visible pellet. One milliliter of 75% reagent-grade ethanol was then added to each tube, followed by centrifugation at 15,000 rpm for 5 min at 4 C. The supernatant was discarded, followed by centrifugation at 15,000 rpm for 5 min at 4 C, and any residual supernatant was also removed and discarded. The Eppendorf tubes were then opened and placed on a heat block set to 55 C for 5 to 10 min (or until no liquid remains). Subsequently, 10 ml of DNase/RNase-free water was then added to the bottom of each tube, and the tubes were then closed and placed back on the heating block for 5 min to dissolve the RNA. This was then followed by centrifugation at 15,000 rpm for 5 min at 4°C. The RNA concentration of the samples was then quantified by nanodrop.

*cDNA synthesis*

After the quantification of mRNA concentration, 30 to 500 ng of mRNA per sample was used to generate complementary DNA (cDNA) using an iScript cDNA Synthesis Kit (20 µl reaction volume) using the following reaction protocol: priming: 5 min at 25 C; reverse transcription: 50 min at 46 C; reverse transcription inactivation: 1 min at 95 C. After cDNA synthesis, the cDNA synthesis reactions were diluted with RNase/DNase‑free water, for example, for every 500 ng of mRNA used, 80 µl of water was added to the cDNA reaction.

### RT-qPCR

Following dilution of cDNA reactions with water, target cDNA amplification was measured using the following reaction components: 5 µl diluted cDNA, 1 µl of 5 µM forward primer stock, 1 µl of 5 µM reverse primer stock, 3 µl of RNase/DNase‑free water, and 10 µl SsoFast EvaGreen Supermix (BioRad, Cat. No. 1725201).

### SDS-PAGE

SDS-PAGE electrophoresis buffer was composed of 24.2 g Tris base, 115.2 g glycine, 8 g SDS, and filled to a final volume of 8 L. Immunoblot transfer buffer was composed of 24.2 g Tris base, 115.2 g glycine, 1.28 l methanol, and filled to a final volume of 8 l. SDS-PAGE gels were cast using mini-PROTEAN spacer plates with 1.5 mm integrated spacers (Bio-Rad Cat. No. 1653312), mini-PROTEAN short plates (Bio-Rad, Cat. No. 1653308), and 15-well 1.5 mm Mini-PROTEAN combs (BioRad, Cat. No. 1653366). Total protein staining: Where indicated, after SDS-PAGE of 2–4 µg of whole‑cell lysate or equal percentages of subcellular fractions described below, gels were stained with Oriole UV-fluorescent stain per the manufacturer’s instructions (Bio-Rad Cat. No. #1610496) to visualize and quantify total protein loading.

### Immunoblotting

2–4 µg of whole‑cell protein lysates were subjected to SDS-PAGE at 200 V for 50 min on 15% polyacrylamide gels. This was followed by electroelution onto polyvinylidene fluoride membranes at 100 V for 50 min under semidry transfer conditions at 4°C. After electroelution, membranes were placed in methanol for 30 s and placed on paper towels to allow methanol to evaporate from the membranes. Membranes were then again placed in methanol for 30 s with gentle rocking, followed by placement into molecular-grade water with gentle rocking again for 30 s, followed by 12 to 16 h at 4°C in PBS supplemented with 0.01% Tween-20 (Sigma-Aldrich, Cat No. A7030) (PBST), 5% BSA (Cat. No. P2287), and the appropriate antibody. Membranes were then washed three times for 15 min in PBST and then incubated for 45 min at room temperature with the appropriate HRP-conjugated anti-IgG or streptavidin-conjugated HRP (Jackson ImmunoResearch Laboratories, Inc) diluted at 1:2000 in 5% BSA dissolved in PBST. Membranes were then washed three times for 15 min with gentle rocking in PBST, subjected to enhanced chemiluminescence imaged using a Bio-Rad ChemiDoc System. Immunoblots were quantified using ImageJ software densitometry (https://imagej.net/ij/download.html).

### Immunocytofluorescence

Slides were fixed with 4% paraformaldehyde in DPBS on ice for 15 min. Slides were then washed three times with ice-cold DPBS followed by permeabilization with 0.5% Triton X-100, 3 mM EDTA for 10 min on ice, and then washed three times with DPBS. Slides were then incubated with primary antibodies (antibodies against TCAP and α-actinin, 1:1000) diluted in 5% BSA in DPBS for 16 h at 4°C in a humidified chamber. Slides were subsequently washed three times with ice-cold DPBS (5 min/wash) and then incubated at room temperature, in the dark for 90 min, with the appropriate fluorophore-conjugated secondary antibodies (ThermoFisher Scientific, 1:1000). Slides were subsequently washed three times with ice-cold DPBS, counterstained for 1 min with DAPI (1:1000), washed three more times with ice-cold DPBS, and then prepared with mounting fluorescence medium and cover slides.

### Antibodies used in this study

TCAP (Abcam: ab133646); GAPDH (EMD Millipore-Sigma: MAB374); Histone H3 (Cell Signaling Technologies, Cat. No. 9715); histone H1.0 (Abcam: ab1790); H3.3 (Abcam: ab176840), Myl2 (ab92721); α-actinin (Millipore-Sigma, Cat. No. A7811).

### Treatment of NRVMs with PE or ISO

NRVMs were first serum-starved for 48 h with serum-free DMEM/F:12 supplemented with penicillin/streptomycin and ITS and then treated with 50 μM PE (Sigma-Aldrich, Cat. No. P6126) dissolved in DMEM/F:12 supplemented with ITS and antibiotics for 2–24 h. Where relevant, culture medium containing PE was replaced with fresh DMEM/F:12 supplemented with ITS and antibiotics. Isoproterenol (Sigma-Aldrich, Cat. No. I6504) dissolved in culture medium was added to each well to a final concentration of 20 μM.

### Hypoxia in vitro

48 h after plating at 200,000 cells/well/mL on 12-wells (Figures. 7, 8A-C, 9A-H), the NRVM culture medium was replaced with DMEM/F:12 supplemented with insulin, transferrin, selenium, and antibiotics. Cultures were then placed in a hypoxia chamber (StemCell Technologies, Catalog # 27310), which was purged with 95% N_2_/5% CO_2_ for approximately 4 min at a pressure not exceeding 4 psi (upper pressure limit of the chamber). Establishment and maintenance of hypoxic conditions were visually confirmed by a yellow coloration of the culture medium due to acidified phenol red.

### Postnatal cardiac maturation time course

Sprague Dawley pregnant dams (Charles River) delivered on ~E17 were monitored daily starting at E19 until birth, during which the animals are maintained on a 12/12 h light/dark cycle. P0 was decided based on sings of recent birth (blood in rat bedding, clumped bedding adhered to damp neonates). Neonates were collected at 12–2 pm PST and sacrificed via decapitation. Excised hearts were briefly washed in ice-cold phosphate‑buffered saline, dried on Kim wipes and weighed, and flash-frozen in Eppendorf tubes. Apical tissue was homogenized with trizol or protein lysis buffer and processed as described above.

### Postnatal hypoxia exposure

Lactating dams with 5–6 pups, from postnatal days 2–8, were maintained either in a control cage exposed to room air (21% O_2_) or in a hypoxia chamber outfitted with a 10% O2/90% N_2_ industrial gas tank (AirGas), Litter size for these experiments was capped at 6 to optimize neonatal access to the lactating dam. Using this experimental setup, gas flow was calculated to be approximately 1.4 ft^3^/hour. It was noted that flow rates below this were found to not support animal survival. Food, water, and bedding were changed every second day, and cages were inspected daily for dead animals, e.g., in Figure 8E-G, one neonate from the hypoxic group was removed as it was found dead during the study. Neonatal hearts were collected as described above.

### Isolation of myocytes via perfusion-digestion and myocyte binucleation measurements

P6-P9 rats were injected with 100 USP units of heparin and then sacrificed by decapitation. The hearts with intact aorta were then cannulated and perfused with 1x Ads buffer containing 2,3-butanedione monoxime (BDM) at 10 mM (Sigma-Aldrich, Cat. No. B0753). The hearts were then perfused with digestion buffer supplemented with BDM and 50 mg/Type 2 Collagenase (Worthington) in 1x Ads for 7–10 min. After sufficient digestion, the hearts were perfused with Kraft-brühe (KB) Buffer supplemented with 10 mM BDM. Digested hearts were placed in ice-cold KB buffer also containing BDM. The atria were removed, and ventricular myocytes were dissociated into solution by trituration with a plastic transfer pipette and filtered (100 µm strainer), followed by centrifugation at 300 g for 5 min. The resulting myocyte pellet was resuspended in KB buffer containing BDM. Myocytes were plated on fibronectin-coated Nun-Tek 2-Chamber slides (ThermoFisher Cat. No. 154461) at ~250,000 cells/well, in parallel with P1 NRVM cultures, and subjected to immunocytofluorescence as described above. Images were taken on a fluorescence microscope using the 20X objective for binucleation counting or 40X for TCAP and a-actinin staining.

## Data Availability

Figure 1A-C: Generated via publicly available data accessible from GEO using accession numbers GSE110604, GSE124008, and GSE102532. The accompanying raw data files of this study were uploaded to Figshare data repository: https://figshare.com/s/ec66cd4725396f11ad95. Figure 1D-H: Raw data presented in supplemental Table S1. Figures 3 and 4: Raw data presented in supplemental Table S2. Figure 6: Raw data presented in supplemental Table S3. Figure 7: Raw data presented in supplemental Table S4. Figure 8: Raw data presented in supplemental table S5. Figure 9: Raw data presented in supplemental table S6.
